# Delays in Temporary and Permanent Pacemakers: Causes and In-Hospital Outcomes

**DOI:** 10.7759/cureus.6953

**Published:** 2020-02-11

**Authors:** Muhammad Irfan, Imran Khan, Kaleem Ullah Bacha

**Affiliations:** 1 Cardiology, Lady Reading Hospital, Peshawar, PAK; 2 Cardiology/Clinical Cardiac Electrophysiology, Lady Reading Hospital, Peshawar, PAK

**Keywords:** temporary pacemaker, permanent pacemaker, delay, causes, outcomes

## Abstract

Background

Temporary pacemakers frequently serve as a bridge to permanent pacemakers, but placement of the latter may be delayed. This study assessed the causes and in-hospital outcomes of patients with delayed placement of permanent pacemakers.

Methods

This retrospective study included all patients admitted to the Emergency Department who underwent temporary transvenous pacing in the Department of Cardiology, Lady Reading Hospital, Peshawar, Pakistan. The duration of hospitalization and the time from temporary to permanent placement were calculated in days. Asystole, infections, cardiac arrest, and death were recorded during the waiting period.

Results

Of the 260 patients who underwent temporary transvenous pacing, 136 (52.3%) were males and 124 (47.7%) were females, with an age range of 46-78 years. Coronary artery disease was prevalent in 34% of the patients. Only 5% of the patients were on arteriovenous (AV) nodal blocking agents, 44% had complete AV block, 22% had sinus node disease, and 14% had slow atrial fibrillation. The cause of high-degree AV block could not be determined in most patients. Most patients with ischemia- and hyperkalemia-induced AV block recovered. AV blocks induced by ischemia and with no known cause were not reversible, with most of these patients receiving permanent pacemakers. Of the 260 patients with high-degree AV block, 165 (63.5%) recovered. The mean waiting time for permanent pacemaker implantation was 8.7 ± 5.4 days. The waiting time was associated with increased infections and adverse hospital course.

Conclusion

A longer waiting period between permanent pacemaker indication and implantation is dangerous, as it is associated with an increased risk of adverse events such as infections, syncope, asystole, malignant arrhythmias, cardiac arrest, and death.

## Introduction

Temporary transvenous pacing (TTvP) is lifesaving in patients with symptomatic arteriovenous (AV) blocks and serves as a bridge to permanent pacemaker (PPM) implantation. TTvP is indicated for various symptoms caused by third-degree AV block, bradyarrhythmias, and life-threatening tachyarrhythmias [1-4]. The increasing need for TTvP has resulted in an increased need for PPM implantation worldwide [5]. Most physicians at tertiary care hospitals in Pakistan lack the expertise for PPM implantation. Moreover, delays are frequent between TTvP and PPM implantation, even in centers where the expertise is available. Delays may be due to the limited financial resources of the patients, a shortage of PPM devices, or catheterization rooms being too busy as these rooms are also used for device implantation. The time from symptom onset to PPM implantation may last from 24 hours to several days [6]. Delays in PPM implantation can increase complication rates as well as patient concern and discomfort.

The literature has reported increased infection rate, and that cardiac arrest due to TTvP leads to displacement and death due to sudden cardiac arrest or arrhythmias [7]. Moreover, the causes and outcomes of delay have not been analyzed in patients awaiting PPM implantation in Pakistan.

## Materials and methods

This study was conducted in the Department of Cardiology of Lady Reading Hospital, Peshawar, Pakistan, the largest public sector hospital with 1,500 beds in Khyber Pakhtunkhwa, a province populated with 35 million people, with patients throughout this province referred to this hospital. The charts of all patients with TTvP admitted through the Emergency Department were retrospectively evaluated. The patients were followed from hospitalization to PPM implantation. Baseline characteristics, including patient age, gender, symptoms, and time of symptom onset, were retrieved from the patients’ charts or the referral slip from another hospital. The indication for PPM was determined by the attending cardiologist. The waiting period was defined as the time, in days, from initial symptom onset to PPM implantation. The duration of hospitalization was also calculated in days. Delays due to comorbidities, including inferior wall ST-segment elevation myocardial infarction and medications such as atrioventricular nodal blocking drugs that were not due to logistic reasons, were also calculated in days. In-hospital outcomes included infection, defined as a recorded fever above 100.3°F, total leukocyte count above 11,000/µL, or start of antibiotic treatment after hospitalization. Patients meeting these criteria at admission were excluded from the study. The delay due to infection was also recorded in days.

In-hospital cardiac outcomes included asystole, defined as a pause lasting more than 3.5 seconds, cardiopulmonary arrest requiring cardiopulmonary resuscitation, sustained or non-sustained ventricular tachycardia, syncope loss of consciousness not due to any other known metabolic cause, and death during the waiting period. Categorical variables were compared using chi-square tests. All statistical analyses were performed using the SPSS Software for Windows, Version 23.0 (IBM Corp., Armonk, NY), with P < 0.05 considered statistically significant.

## Results

Of the 260 patients who presented with high-degree AV block, 136 (52.3%) were males and 124 (47.7%) were females, with a patient age range of 46-78 years. Most patients were hypertensive, with 34% having coronary artery disease. Only 5% of the patients were on AV nodal blocking drugs, with most of these taking beta-blockers (Table 1).

**Table 1:** Baseline demographic and clinical characteristics of patients with high-degree atrioventricular block Results are reported as mean ± SD or number (%) SD, standard deviation

Baseline characteristics	N = 260
Age (years), mean ± SD	62 ± 16
Male, n (%)	136 (52.3%)
Comorbidities, n (%)	
Hypertension	84 (33%)
Diabetes	55 (21.2%)
Hypothyroidism	9 (3.6%)
Coronary artery disease	87 (34%)
Medications, n (%)	13 (5%)
Beta-blockers	7 (2.7%)
Calcium channel blockers	3 (1.1%)
Digoxin	1 (0.03%)
Amiodarone	1 (0.03%)
Ivabradine	1 (0.03%)
Biochemical profile, mean ± SD	
Serum potassium, mEq/L	4.8 ± 2.2
Serum creatinine, mg/dL	1.4 ± 2.3
Troponin I, ng/mL	3.2 ± 6.2

**Figure 1:**
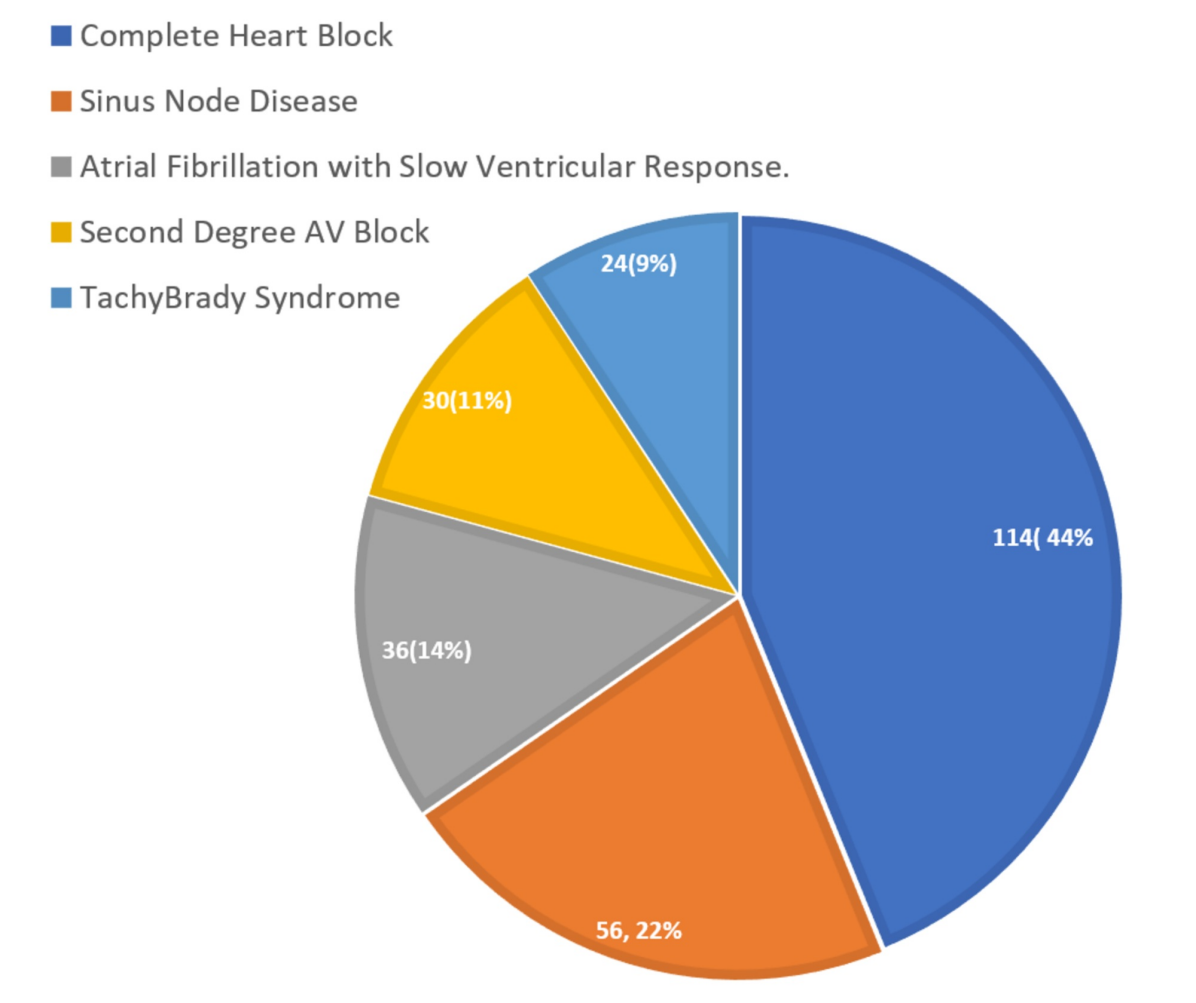
Indications for pacemaker implantation AV, atrioventricular

Of the patients with indications for TTvP due to symptomatic high-degree AV nodal block, 44% had complete AV block, 22% had sinus node disease, and 14% had slow atrial fibrillation (Figure 1).

The causes of high-degree AV block could not be determined in most patients. Most patients with AV block induced by drugs or hyperkalemia recovered. In contrast, AV blockages induced by ischemia or with no known cause were not reversible, with most of these patients requiring PPM implantation (Table 2).

**Table 2:** Association between the type of pacemaker and the etiology of high-degree AV block AV, atrioventricular; CKD, chronic kidney disease

Type of pacemaker	Cause of AV block				P-value
	Drugs	Ischemia	CKD hyperkalemia	Unknown	
Temporary pacemaker only	10	59	33	63	0.7
Permanent pacemaker only	0	7	0	27	
Temporary followed by permanent pacemaker	3	26	8	24	

Of these 260 patients with high-degree AV block, 165 (63.5%) recovered and were discharged, whereas 95 (36.5%) underwent PPM implantation (Table 3).

**Table 3:** Temporary and permanent pacemaker implantations and length of hospital stays AV, atrioventricular; PPM, permanent pacemaker

Implantation and waiting period data	N (%)
Temporary pacemaker implanted only for reversible high-degree AV block	165 (63%)
Elective PPM implantation for stable high-degree AV block	40 (15.3%)
Temporary pacemaker followed by permanent pacemaker for high-degree AV block	55 (21%)
Time from hospitalization to PPM implantation (days)	8.7 ± 5.4 days
Waiting period due to co-morbidities (days)	3.2 ± 4.1 days
Waiting period due to infection during the waiting period (days)	7.2 ± 3.9 days
Waiting period due to lack of logistics (days)	5.6 ± 3.7 days

**Figure 2:**
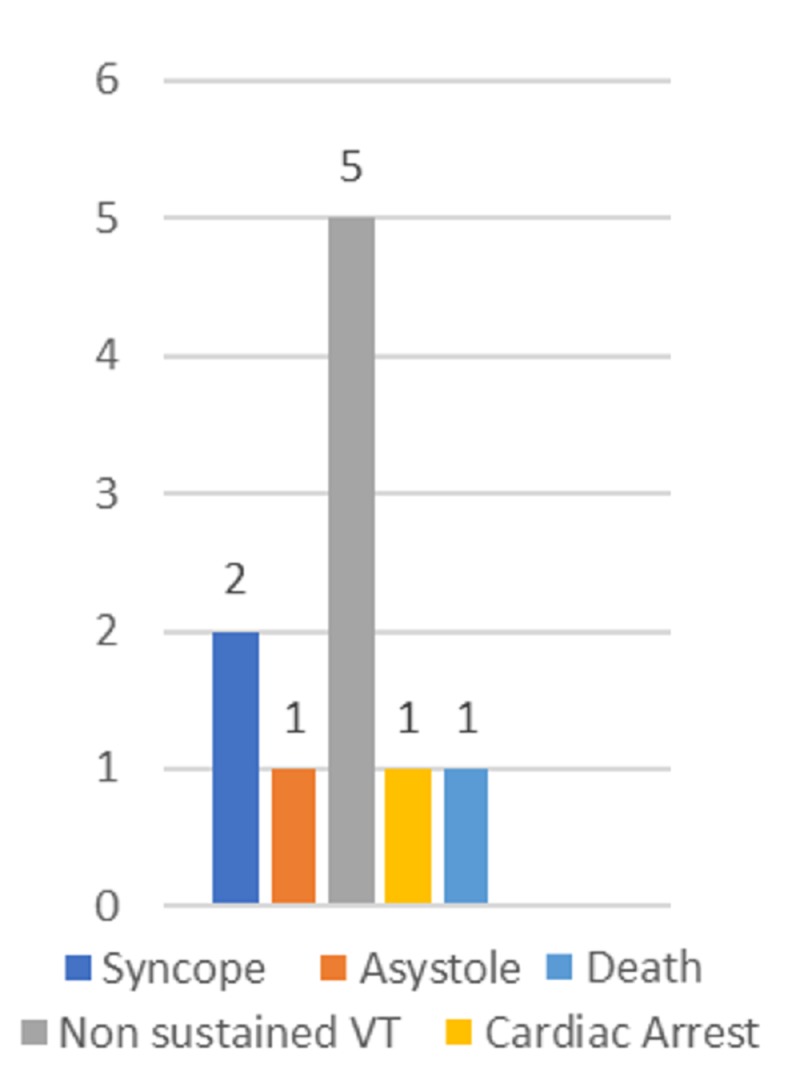
Overall adverse events during hospital stay in patients implanted with temporary transvenous pacemakers followed by permanent pacemakers (n = 55). VT, ventricular tachycardia

The mean waiting time for PPM implantation was 8.7 ± 5.4 days. The main reason for the delay was the unaffordability of the device. Delay in the device implantation led to infection due to temporary venous lead, which is exposed to the environment and is a source of infection. PPMs are not implanted until infection is treated. Thus, infection was another cause for the delay. The delay time from TTvP to PPM implantation was associated with increased infection and adverse hospital course (Figure 2).

## Discussion

The delay from TTvP to PPM implantation is common in developing countries, including Pakistan, as the populations of these countries cannot afford quality treatment. Most of the patients who had a reversible cause for AV block had ischemia or hyperkalemia. In idiopathic cases, we presumed the cause to be conduction tissue disease with paroxysmal AV block. This study found that the delay in PPM implantation was associated with increased morbidity. Reasons for delay included associated comorbidities and lack of logistic support, including the absence of available catheterization laboratories and PPM devices. These results are consistent with many other worldwide studies [8], which found that increased waiting period in the hospital not only increased in-hospital adverse outcomes, such as asystole and arrhythmias, but also increased the likelihood of in-hospital infections. Moreover, the risk of infection was higher in patients with a prolonged hospital stay, further increasing the waiting period. Most infections were documented during the hospital stay. Infection further increased the mean waiting period of 7.2 days. Comorbidities were found to delay PPM implantation by 3.2 days, similar to previous findings [9]. In our study, TTvP electrode catheters were inserted until PPMs were implanted. Daily electrocardiogram (ECG) and temporary pacemaker threshold are checked for pacemaker-dependent patients in our institution. If the threshold is high or there is evidence of loss of capture on ECG, the lead position is checked under a fluoroscope. Even then, displacement of the temporary pacemaker wire does occur, causing in-hospital arrhythmias and asystole, especially in patients with a longer waiting period. Patients awaiting PPM implantation have significant morbidity and mortality rates, emphasizing the need to minimize these delays [10]. Adverse events following delay included infections and even cardiac arrest due to heart block. Temporary pacing wires are associated with substantial rates of complications and morbidity [11], which may be avoided by implanting a PPM as soon as indicated. Our study reported death and life-threatening arrhythmias, which could have been avoided if PPM was implanted in time. Our study has certain potential limitations. Coronary angiography to exclude concomitant coronary artery disease could not be performed in our patients. We could not document whether infection occurred before admissions or after TTvP lead insertion, but we believe that the infection could have been avoided if PPM was implanted soon after admission.

## Conclusions

A delay between PPM indication and implantation is dangerous, as it is associated with an increased risk of adverse events such as infections, syncope, asystole, malignant arrhythmias, cardiac arrest, and death. Facilities for PPM implantation should be available 24 hours per day in the hospital. This will reduce not only patient morbidity but also the cost of hospitalization.
